# Diagnosis and Management of IUGR in Pregnancy Complicated by Type 1 Diabetes Mellitus

**DOI:** 10.1007/s11892-016-0732-8

**Published:** 2016-03-16

**Authors:** Paweł Gutaj, Ewa Wender-Ozegowska

**Affiliations:** Department of Obstetrics and Women’s Diseases, Poznan University of Medical Sciences, Polna 33, 60-535 Poznan, Poland

**Keywords:** Intrauterine growth restriction, IUGR, Small for gestational age, SGA, Type 1 diabetes, Vasculopathy

## Abstract

This review discusses available literature on the diagnosis and management of intrauterine growth restriction (IUGR) in women with type 1 diabetes. IUGR is diagnosed when ultrasound-estimated fetal weight is below the 10th percentile for gestational age. IUGR diagnosis implies a pathologic process behind low fetal weight. IUGR in pregnancy complicated by type 1 diabetes is usually caused by placental dysfunction related to maternal vasculopathy. Prevention of IUGR should ideally start before pregnancy. Strict glycemic control and intensive treatment of nephropathy and hypertension are essential. Low-dose aspirin initiated before 16 gestational weeks can also reduce IUGR risk in women with vasculopathy. Umbilical and uterine artery Doppler studies can guide diagnosis and surveillance of fetuses with IUGR. Decisions regarding the timing of delivery should be based on assessment of umbilical artery Doppler. The risk of prematurity and impaired fetal lung maturation should always be considered, especially in fetuses younger than 32 weeks.

## Introduction

Fetal growth is a result of complex interactions between the genetic growth potential of the fetus and the impact of the maternal intrauterine environment. Causes of intrauterine growth restriction (IUGR) are generally classified as maternal, fetal, or placental. However, such distinction is rather theoretical, as these factors often overlap each other.

Impaired fetal growth not only results in elevated risk for short-term complications, with intrauterine fetal death being the most severe, but low birth weight also predisposes the child to cardiovascular disease and type 2 diabetes mellitus in the future [1, 2].

IUGR is a common cause of iatrogenic prematurity, as a manifestation of ischemic placental disease—a syndrome of placental insufficiency found in several pregnancies terminated prematurely due to fetal conditions.

IUGR is diagnosed when ultrasound-estimated fetal weight is below the 10th percentile for gestational age. A diagnosis of IUGR implies a pathologic growth restriction responsible for low fetal weight [3–5]. Small for gestational age (SGA) is another term that defines a fetus with an estimated weight below the 10th percentile; however, the term SGA does not imply a pathological condition causing low fetal weight. Approximately 70 % of SGA fetuses are constitutionally small, meaning they are small but healthy. The remaining are fetuses with IUGR, which are at high risk for perinatal complications and therefore require more intensive surveillance [6•].

Fetal growth abnormalities are frequently seen in diabetic pregnancy, with large for gestational age (LGA) being the most typical one. LGA has been widely studied and seems to be associated most frequently with maternal hyperglycemia, although other factors such as gestational weight gain and maternal lipids may also play a role [7–12]. However, diabetic pregnancy can also be associated with restricted fetal growth. Long-standing and poorly controlled type 1 diabetes represents a wide spectrum of clinical conditions, mostly related to diabetic vasculopathy. Since the placenta is an organ composed almost entirely of vessels, maternal vasculopathy can be linked to placental dysfunction and subsequent fetal growth restriction [13–15].

The incidence of type 1 diabetes is rising at an alarming rate, particularly in younger age groups. Although disease outcomes have been improving over the past decades, resulting in a reduction in total mortality, these beneficial trends start to disappear for the incidence of nephropathy and proliferative retinopathy at 20 years of diabetes duration [16]. Thus, it may be expected that an increasing number of women with diabetic vascular disease are now, or soon will be, entering childbearing age. IUGR could therefore become one of the major pregnancy complications exacerbated by type 1 diabetes.

This review discusses available literature on the diagnosis and management of IUGR in women with type 1 diabetes. However, data on IUGR in type 1 diabetic pregnancy is limited and there are no randomized controlled trials on the management of IUGR in this group of patients. This significantly limits the strength of the evidence. Therefore, we discuss existing guidelines for a normal population in the context of diabetic pregnancy and focus on expert opinion as well as our personal experience regarding the management of diabetic pregnancy.

## Pathogenesis of IUGR in Pregnancy Complicated by Type 1 Diabetes—the Key Role of Diabetic Vasculopathy

Many factors can potentially interfere with genetically predetermined fetal growth causing its restriction. In this section, we provide an overview of maternal and placental factors related to diabetes that cause IUGR.

The incidence of type 1 diabetes peaks in 10–14 year olds and is rising at an alarming rate worldwide, particularly in younger age groups (0–4 years) [17]. Many of these girls are reaching childbearing age as women with long-standing diabetes, frequently complicated by vasculopathy.

Metabolic alterations associated with diabetes impact the development and function of utero-placental and feto-placental units from the beginning of pregnancy. Fetal growth in type 1 diabetic pregnancy might be altered in two opposing ways. Maternal hyperglycemia stimulates fetal overgrowth, while maternal vasculopathy can be associated with placental insufficiency leading to altered nutrient transport and subsequent IUGR.

### Histopathological Studies

Several histopathological changes can occur in the placenta in response to a diabetic environment [18]. Although some of them are not specific for the diabetic placenta, maternal arterial malperfusion, distal villus maldevelopment, and fetal vasculitis might be considered characteristics for diabetic placentas. Maternal arterial malperfusion occurs as a consequence of inadequate and incomplete invasion of the trophoblast and deficient spiral artery transformation [19]. Moreover, unremodeled uterine arteries can additionally present features of decidual vasculopathy. It has been previously shown that almost a quarter of type 1 diabetic placentas harbored decidual vasculopathy [20]. These pathological changes are associated with IUGR, particularly in preeclamptic women [19, 21]. Women with type 1 diabetes are at substantially higher risk of preeclampsia. Furthermore, the risk increases with longer diabetes duration, with the highest rates reported in women with diabetic nephropathy, placing them at the highest risk of developing IUGR [22]. Distal villus maldevelopment is a term representing a group of histopathological findings including distal villous hypoplasia, distal villous immaturity, chorangiosis, and dysmorphic villi. These changes are associated with the reduction of functional villus surface area and can be observed in a significant proportion of IUGR placentas [23, 24]. Fetal vasculitis is a common histopathological finding in diabetic placentas and might be a marker of fetal inflammatory response [20, 25]. The role of fetal vasculitis in IUGR remains unknown; however, some authors reported an association between different markers of inflammatory state and IUGR [26, 27].

### Biochemical Studies

Abnormalities in placental angiogenesis due to maternal microangiopathy have their roots early in pregnancy. It has been demonstrated that placental growth factor (PlGF) concentration dynamics is impaired in women with type 1 diabetes whose fetuses later develop SGA. While PlGF serum concentration doubled between the first and second trimesters in women with type 1 diabetes who delivered newborns with normal growth, there was no physiological increase in PlGF in the SGA group. Importantly, the majority of women from the SGA group had some form of diabetic vasculopathy [13]. Other interesting findings come from a study on markers of endothelial dysfunction. Zawiejska et al. observed elevated maternal serum concentration of vascular cell adhesion molecule-1 (VCAM-1) in early gestation and low levels in late gestation in women with type 1 diabetes who gave birth to SGA newborns. The authors hypothesized that observed changes may be a manifestation of endothelial injury in early gestation followed by placental dysfunction in late gestation. Early vascular dysfunction was also proposed as a major factor involved in preeclampsia in women with type 1 diabetes. The authors of this study demonstrated significantly increased first trimester serum levels of VCAM-1 and intercellular adhesion molecule-1 (ICAM-1) in the preeclamptic group [28].

### Doppler Ultrasound Findings

Although placental histopathological findings provide substantial and clinically relevant information, these data are always retrospective and represent pathological processes and adaptations that have been taking place sometimes many weeks before delivery. Doppler ultrasound enables the real-time assessment of placental and fetal circulation and has been widely used in the monitoring of high-risk pregnancies. Moreover, abnormal umbilical and uterine Doppler velocity waveforms in IUGR have been correlated with abnormal placental pathology [29–31]. Efficient utero-placental and feto-placental circulation is crucial for the maintenance of physiological fetal growth. Pietryga et al. demonstrated significantly increased uterine artery vascular impedance in pregnant women with type 1 diabetes with vasculopathy [32]. Furthermore, increasing uterine artery scores were associated with an increasing proportion of SGA newborns. In this study, abnormal uterine Doppler was almost exclusively seen in women with classes R/F retinopathy and nephropathy, according to White [33]. Women with uncomplicated diabetes or isolated retinopathy had relatively normal Doppler results. The proportion of abnormal flow in umbilical arteries was significantly lower and unrelated to vasculopathy. This suggests that impairment of utero-placental circulation is a primary pathology in the placentas of women with severe vasculopathy and changes in the umbilical artery may occur later as an adaptation to reduced utero-placental perfusion. Impairment of placental function in diabetic vasculopathy has been directly confirmed in a study by Salvesen et al. It was conducted in a cohort of 41 women with type 1 diabetes undergoing cordocentesis up to 24 h before delivery. The authors found that all fetuses of women with nephropathy and hypertension had significantly lower umbilical vein pH than the normal mean for gestation (<5th percentile) [34].

## Diagnosis of IUGR

### Diagnostic Approach

Because IUGR is associated with unfavorable perinatal outcomes and therefore has a strong impact on the management of pregnancy, it should be clearly distinguished from constitutional small for gestational age (cSGA). The most widely used definition of IUGR is based on the estimated fetal weight (EFW) below the 10th percentile [3–5]. However, according to the Royal College of Obstetricians and Gynecologists (RCOG), the diagnosis of IUGR can also be made on the basis of fetal abdominal circumference (AC) below the 10th percentile [4]. The brief summary of diagnostic criteria as well as important aspects on the management of IUGR is summarized in Table 1. It should be mentioned that percentile charts used for these determinations were developed based on the healthy population. Persson et al., in their large population-based cohort study, showed that distribution of birth weight (BW) of offspring born to the mothers with type 1 diabetes was significantly wider and shifted to the right of the normal reference. Moreover, newborns of the mothers with type 1 diabetes had higher mean BW despite significantly lower mean gestational age (GA) at birth [35]. Similar findings have been demonstrated by others in smaller cohorts [36, 37]. However, Howart et al. showed that such distribution is rather a characteristic for uncomplicated type 1 diabetic pregnancy. These authors demonstrated that customized BW percentiles of newborns of women with vascular disease (defined as the presence of at least one of the following: diabetic retinopathy, diabetic nephropathy, or preexisting hypertension) were bimodally distributed [38]. The authors concluded that coexistence of diabetes and vascular disease produces an even more unfavorable intrauterine environment, which may falsely “normalize” the growth of fetuses destined to develop macrosomia. One may suggest that among these fetuses, some could be growth restricted due to impaired placental function and “normalized” by maternal hyperglycemia. These facts raise the question whether the diagnosis of IUGR in women with type 1 diabetes is appropriate using charts based on a healthy population. This question still remains open, as there is no evidence in the literature to support different diagnostic approaches. Therefore, the diagnosis of IUGR in women with type 1 diabetes should be based on the same criteria as for the reference population. Nonetheless, special attention should be paid to fetuses with borderline estimated fetal weight, as the risk for false negative diagnosis may be increased due to changes in their weight distribution. In suspicious or questionable cases, uterine and umbilical artery Doppler ultrasound may provide useful diagnostic information, especially when IUGR is of placental origin in women with diabetic vasculopathy [32, 39].

**Table 1:** A brief summary of the statements of ACOG, RCOG, and SOGC on the management of pregnancy complicated by IUGR

	ACOG	RCOG	SOGC
Definition	EFW <10th percentile	EFW <10th percentile or AC <10th percentile	EFW <10th percentile
Main method for the follow-up	Umbilical artery Doppler	Umbilical artery Doppler	Umbilical artery Doppler
Antenatal corticosteroids	Single course when there is a significant risk of delivery <34 weeks of gestation	Single course until 35 + 6 weeks of gestation when preterm delivery is anticipated	Single course when there is a significant risk of delivery <34 weeks of gestation
Low-dose aspirin for the prevention of IUGR	Lack of evidence for its preventative effects for IUGR Recommended for women at high risk of preeclampsia	Lack of evidence for its preventative effects for IUGR Recommended for women at high risk of preeclampsia	High risk patients, including women with pregestational diabetes and women with a previous history of IUGR or preeclampsia

Data from [3–5]

### Importance of Pregnancy Dating

Accurate pregnancy dating is essential for the diagnosis of IUGR. However, estimation of GA based on the last menstrual period (LMP) might be inadequate in a significant proportion of women with type 1 diabetes due to frequent menstrual irregularities [40, 41].

Measurement of crown-rump length (CRL), optimally between weeks 9 and 13 of gestation, provides the most reliable basis for further evaluation of fetal growth and should be precisely documented in women with type 1 diabetes [42, 43].

### Early Detection of Women at Risk

Detection of patients at risk of IUGR should ideally start early in the pregnancy. In addition to traditional medical and obstetric risk factors for IUGR, women with type 1 diabetes should be screened for vascular disease, primarily nephropathy [14, 38]. Notably, not only overt nephropathy but also microalbuminuria has been associated with IUGR [15]. Despite the known association between diabetic retinopathy and adverse pregnancy outcomes, there are no studies directly linking diabetic retinopathy to IUGR [44, 45]. One study reported no difference in the incidence of fetal growth restriction between women with microvascular complications (retinopathy and nephropathy, analyzed together) and those without them [46]. This might be attributed to the significant improvements in the quality of care in pregnant women with type 1 diabetes, particularly intensification of antihypertensive treatment [47•].

Diabetes, especially when uncontrolled early in pregnancy, increases the risk of fetal structural anomalies which may also manifest as impaired fetal growth [48, 49]. IUGR fetuses should therefore undergo detailed anatomy scans involving echocardiography.

## Management of IUGR

IUGR increases the risk of adverse pregnancy outcomes including preterm delivery, intrauterine fetal death (IUFD), neonatal death, low APGAR scores, and abnormal neurodevelopment of the child [50, 51]. Thorough prenatal surveillance and appropriate timing of delivery can reduce the risk of perinatal mortality and morbidity in women with type 1 diabetes. Women with pregnancies complicated by type 1 diabetes and IUGR should be managed by maternal-fetal medicine specialists experienced in the field of diabetic pregnancy at a perinatal center with access to a neonatal intensive care unit.

### Ultrasound Surveillance

Ultrasound biometry is essential for the diagnosis of IUGR due to its superiority in clinical weight estimation using fundal height measurements. Therefore, it seems reasonable to perform ultrasound biometry in all women with risk factors for fetal growth abnormalities, including those with diabetes. IUGR associated with diabetic vasculopathy and resultant placental insufficiency is typically asymmetric and does not manifest before the third trimester [52]. Symmetric growth restriction diagnosed earlier in the second trimester raises the suspicion of genetic disorders or congenital malformations and standard management should be offered to these women.

Subsequent serial ultrasound measurements provide further information on fetal growth pattern, which might be altered in women with type 1 diabetes [52]. The optimal intervals between scans have not been determined and should be based on individual clinical judgment. However, it is suggested that repeated measurements should be performed at least 3 weeks apart to reduce the possibility of a false positive diagnosis [3, 4]. There is no evidence to support more frequent fetal biometry in women with type 1 diabetes and IUGR.

Amniotic fluid volume (AFV) assessment is a routine part of ultrasound scanning during pregnancy. Although, oligohydramnios is associated with SGA and perinatal mortality, its predictive value for individual risk for the outcome is poor [53]. Thus, AFV measurement should not be used as the only method of monitoring IUGR fetuses. However, in conjunction with Doppler studies or as a part of biophysical profiles (BPP), it can provide more clinically relevant information on fetal well-being. AFV can be assessed by subjective (based on the examiner’s visual assessment) and objective (amniotic fluid index [AFI] and single deepest pocket [SDP]) methods. It is recommended to measure AFV objectively when either subjective measurements suggest decreased AFV or in patients with higher risk of IUGR. Comparison of AFI and SDP revealed that AFI was associated with higher rates of false positive diagnosis of oligohydramnios which may lead to unnecessary labor induction [54]. Thus, SDP measurement may be preferred over AFI, especially in preterm pregnancies.

Although there are no randomized trials examining the utility of ultrasound Doppler surveillance in pregnancy complicated by type 1 diabetes, existing evidence from observational studies support this method as the best for detection of fetal distress in high-risk pregnancies, including IUGR.

Doppler ultrasound directly reflects impairment of utero-placental and feto-placental circulation which is, in the case of IUGR, related to maternal vasculopathy [32, 39]. In addition to its essential role in determining the cause of fetal growth restriction (placental insufficiency vs. constitutional SGA/other causes), umbilical Doppler velocimetry has been widely recommended as a main method for the follow-up of IUGR fetuses [3–5]. In pregnancy complicated by type 1 diabetes, umbilical artery Doppler velocimetry was found to be superior to non-stress test and BPP in identifying fetuses at risk of adverse outcome [55]. In normal circumstances, in the second part of pregnancy, umbilical and uterine arteries are low-resistance vessels. In cases of placental dysfunction, both vessels can present features of increasing flow resistance. These alterations are manifested in Doppler as increasing pulsation index (PI), the presence of end-diastolic notches in uterine arteries, and increasing PI in umbilical arteries. Increasing resistance in umbilical arteries can cause severe hemodynamic changes which can be further manifested as absent end-diastolic velocity (AEDV) and reversed end-diastolic velocity (REDV).

### Biophysical Profile (BPP) and Fetal Heart Rate Monitoring

The number of studies on the utility of BPP and fetal heart rate monitoring (FHRM) in pregnancy complicated by type 1 diabetes is limited [34, 55, 56]. It was shown that both BPP and FHRM had lower predictive value of adverse pregnancy outcomes including fetal growth restriction than umbilical artery Doppler [55] and their results were not predictive for fetal acidemia [34]. The low predictive value of abnormal BPP has been confirmed in a prospective study using multiple BPP from 28 weeks until delivery [56]. Nonetheless, the high predictive value of normal BPP makes it a more valuable tool for surveillance of fetuses at high risk of IUFD, including these with IUGR. However, it should be remembered that diabetes may have an impact on the results of BPP as maternal hyperglycemia was found to increase both fetal respiratory movements and AFV [57, 58]. Furthermore, sustained hyperglycemia (>120 mg/dL) was associated with a decrease in fetal movements in healthy women [59]. No significant changes in fetal body and respiratory movements were observed during hypoglycemia [60].

### Maternal Perception of Fetal Movements

Decreased fetal movements (DFM) perception in the third trimester has been associated with increased rates of IUGR, emergency cesarean section (CS), and IUFD [61]. There are no separate guidelines on the management of DFM in women with type 1 diabetes. Nonetheless, reports of DFM should always prompt further investigation, including confirmation of fetal heart rate and weight estimation by ultrasound. If the fetus is found to be IUGR, umbilical and uterine Doppler should be considered due to the high risk of placental dysfunction, primarily in women with vasculopathy [32].

### Diet and Physical Activity

There is no evidence that changes in diet or supplement use can prevent or treat IUGR. Thus, individualized medical nutrition therapy (IMT) for pregnant women with type 1 diabetes is indicated. IMT is aimed at achieving glycemic targets and maintaining appropriate gestational weight gain [62].

There is no evidence to recommend bed rest as a method of either prevention or treatment of IUGR [63]. However, strenuous physical activity should be avoided due to risk of hypoglycemia.

### Glycemic Targets and IUGR

There is no evidence to support the use of individualized glycemic targets in pregnant women with type 1 diabetes and IUGR. Nonetheless, Langer et al. showed in women with gestational diabetes mellitus (GDM) that had low levels of mean glycemia throughout pregnancy (<86 mg/dL) were associated with a significant increase in SGA in comparison to healthy controls (20 vs. 11 %) [64]. To the authors’ knowledge, there are no similar studies in women with type 1 diabetes and IUGR. However, it could be hypothesized that too strict glycemic control in women with already impaired placental function and diminished nutrient transport to the fetus could cause further restriction of fetal growth with all associated complications [65].

### Antenatal Corticosteroids

IUGR is associated with significantly increased rates of preterm deliveries, primarily iatrogenic. Diabetes is not considered a contraindication for the administration of corticosteroids for fetal lung maturation, but this procedure should be precisely planned with increased dosages of insulin [66]. There is no full consensus on the time window when corticosteroids should be given in pregnancies complicated by IUGR. The American College of Obstetricians and Gynecologists (ACOG) and the Society of Obstetricians and Gynaecologists (SOGC) recommend a single course of corticosteroids when there is a significant risk of delivery <34 weeks of gestation [3, 5]. The RCOG recommends that in pregnancy with fetal growth restriction and anticipated preterm delivery, a single course of corticosteroids should be given until 35 + 6 weeks of gestation [4].

However, the strongest evidence exists for supporting the use of corticosteroids until 34 + 6 weeks [67]. Furthermore, bearing in mind that high doses of potent corticosteroids (betamethasone or dexamethasone) can significantly deteriorate maternal glycemic control, it seems reasonable to recommend stimulation for fetal lung maturation until 34 + 6 weeks in women with type 1 diabetes. Treatment with corticosteroids should be followed by appropriate insulin dose adjustment and frequent glycemic monitoring [68, 69].

### Timing and Mode of Delivery

The aim of intensive surveillance of the fetus with IUGR is to detect the safest time for delivery. This is often difficult for even the most experienced obstetrician, especially in cases of fetal prematurity. Thus, several factors should be taken into careful consideration while making a decision on timing and mode of delivery for IUGR fetus. Management always depends on the etiology of IUGR. In women with type 1 diabetes, IUGR is typically of placental origin. Undoubtedly, this is the most difficult to manage and most unpredictable type of IUGR, especially in women with nephropathy and preeclampsia. In such cases, the decision on timing of delivery is often based on either signs of acute fetal distress, deterioration of maternal state, or both. There are several tools for fetal surveillance; however, only some have been proven to predict neonatal outcomes. None of them have been studied exclusively in women with type 1 diabetes and IUGR in the context of timing of delivery. Doppler velocimetry is considered the most sensitive method to detect risk of fetal death. Although several vessels can be examined during this procedure, only umbilical artery velocimetry has been found to decrease the likelihood of perinatal deaths in IUGR [70]. Therefore, it is recommended to base the decision regarding timing of delivery on the results of umbilical artery Doppler studies (absent or reversed end-diastolic velocity [AREDV]) [3, 5, 70]. Nonetheless, GA is the major determinant of fetal survival in IUGR diagnosed before 33 weeks [71]. RCOG recommends that in fetuses with AREDV detected prior to 32 weeks, delivery should be considered when either ductus venosus (DV) abnormalities or umbilical vein (UV) pulsation is found [4].

The IUGR itself is not an indication for CS. However, a significant number of IUGR fetuses is delivered via urgent CS due to acute signs of fetal distress demonstrated either before the onset of spontaneous contractions (umbilical artery AREDV, UV pulsation, DV abnormalities) or intrapartum (late or variable decelerations, loss of fetal heart rate variability). IUGR of placental origin is a manifestation of chronic fetal distress, and the fetus may already be acidotic. Blood flow throughout the placenta decreases during contractions, which may result in metabolic decompensation of the fetus. Thus, it seems reasonable to recommend the use of continuous FHRM during labor in women with type 1 diabetes and IUGR from the onset of contractions [4]. Vaginal delivery of a fetus with umbilical artery AREDV is not recommended and CS is necessary even in very premature periods [4].

### Maternal Interventions and Preventive Strategies

IUGR due to placental insufficiency is a progressive and irreversible disease. Except for intensive prenatal surveillance aimed at establishing appropriate timing of delivery, there are no perinatal interventions proven to be associated with improved outcomes of IUGR fetuses. Therefore, the question arises whether IUGR might be somehow prevented in women with type 1 diabetes. Although limited conclusions can be made regarding the prevention of IUGR in women with type 1 diabetes complicated by vasculopathy, pregnancy planning and appropriate management of vascular complications and hypertension could play an important role. While poor glycemic control in the second part of pregnancy stimulates fetal overgrowth, it can exert the opposite effects in early pregnancy by affecting early placental development. Evidence supporting this association comes from studies linking poor glycemic control in early pregnancy to preeclampsia [72, 73]. IUGR is one of the major complications of preeclampsia, and there is a strong evidence that they share common pathophysiology [74–76]. Thus, interventions that have been found to reduce the risk of preeclampsia can also reduce the risk of IUGR. Low-dose aspirin (50–150 mg) administered at or before 16 weeks of pregnancy has been found to reduce the risk of both [77]. However, there is no consensus on the use of aspirin for prevention of IUGR. The SOGC recommends low-dose aspirin use in high-risk patients, including women with pregestational diabetes and women with a previous history of IUGR or preeclampsia [5]; however, the ACOG and RCOG do not due to lack of evidence for its preventative effects for IUGR [3, 4]. The ACOG and RCOG do, however, recommend its use in patients at high risk of preeclampsia. It is well known that the presence of even mild diabetic nephropathy is one of the strongest determinants of preeclampsia [22, 78, 79]. It is therefore reasonable to recommend low-dose aspirin from 12 gestational weeks for women with type 1 diabetes with vascular disease, primarily nephropathy, for the prevention of preeclampsia and associated complications, including IUGR [47•]. Moreover, early introduction of intensive antihypertensive treatment in women with type 1 diabetes with nephropathy may alleviate the severity of preeclampsia and increase the chances for normal fetal growth [14, 80]. Treatment should be initiated when blood pressure (BP) exceeds 135/85 mmHg or urinary albumin excretion exceeds 300 mg/24 h. Less rigorous therapeutic targets (BP >140/90 mmHg or albuminuria >2000 mg/24 h) have been associated with an increased rate of preeclampsia and preterm delivery [80].

## Conclusion

IUGR in pregnancy complicated by type 1 diabetes is usually the result of placental dysfunction related to maternal vasculopathy. Strict glycemic control and intensive hypertension treatment before and during pregnancy can potentially reduce the risk of IUGR in women with type 1 diabetes by improving placental function. Low-dose aspirin initiated at 12 weeks of gestation may be beneficial for women with type 1 diabetes at risk of IUGR, primarily those with diabetic nephropathy. Management of IUGR should focus on early diagnosis and intensive prenatal surveillance using ultrasound. Umbilical and uterine artery Doppler can be useful in the diagnosis and monitoring of fetuses with IUGR. Decisions regarding the timing of delivery should be based on the assessment of umbilical artery Doppler (the presence of AREDV). However, in fetuses with AREDV detected prior to 32 weeks, delivery should be considered when either DV abnormalities or UV pulsation is found. When preterm delivery is anticipated, antenatal steroids should be administered until 34 + 6 weeks of gestation.
